# Frequency Selective Properties of Coaxial Transmission Lines Loaded with Combined Artificial Inclusions

**DOI:** 10.1155/2014/731947

**Published:** 2014-01-23

**Authors:** Francisco Falcone, Javier Gil

**Affiliations:** Departamento de Ingeniería Eléctrica y Electrónica, Universidad Pública de Navarra, Campus Arrosadıa, 31006 Pamplona, Spain

## Abstract

The properties of a modified coaxial transmission line by periodic inclusions will be discussed. The introduction of split ring resonators, conductor stubs, air gaps, and combination of these gives rise to new frequency selective properties, such as stopband or passband behavior, observable in planar as well as volumetric metamaterial structures. These results envisage new potential applications and implementation of devices in coaxial transmission line technology.

## 1. Introduction

The inclusion of periodic elements loading a transmission line or a volumetric structure such as frequency selective surfaces has been of interest for over the past 60 years. The initial applications of artificial dielectrics and periodic structures, for the implementation of lenses to enhance radar technology [1], evolved to photonic bandgap and electromagnetic bandgap structures from the Infrared frequency range [2] and scaled to the RF and microwave frequency range [3, 4]. In the late 90s, the split ring resonator (SRR) was proposed as an element with a strong magnetic response, implemented with conventional conductors [5]. The combination of these SRR elements with thin wire media gave rise to a so called left-handed metamaterial structure, characterized by exhibiting antiparallel phase and group velocity and E-field, H-field, and k-vectors forming a left-handed triad instead of a conventional right-handed one [6, 7]. The exploration of these structures gave rise to the implementation of a broad range of devices in different configurations, from free space to closed and open waveguides [8–15].

One of the most popular transmission lines employed in communication systems is coaxial cables. This is due to the fact that it is completely shielded, exhibiting high interference protection, and that it supports a transverse electromagnetic mode as the fundamental propagating mode. This implies the lack of a cut-off frequency, which allows among other things the simultaneous transmission of information signals as well as DC or AC power, which enables powering the remote end of the communication link, as applied, for example, in Power over Ethernet devices or in terrestrial microwave communication links. In this sense, the application of inclusions in order to tailor the frequency response of a coaxial transmission line can be of interest in this type of applications. This concept has been extended in order to potentially implement devices such as power dividers, based on a coaxial transmission line loaded with SRR elements as well as with capacitive loaded strips which exhibit frequency selective response [16].

**Figure 1:**
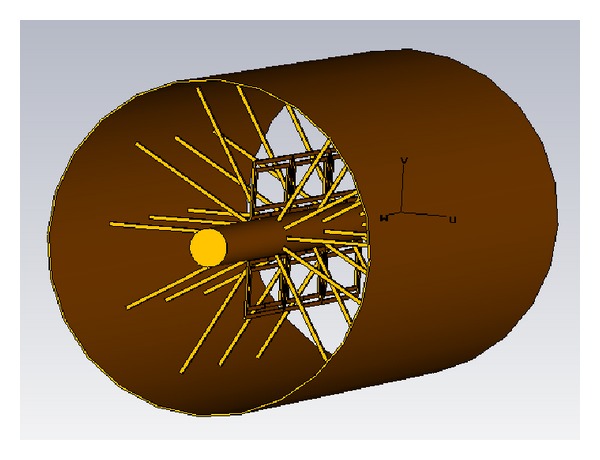
Schematic view of a coaxial transmission line loaded with radial stubs and Split Ring Resonators.

In this work, an alternative configuration in order to implement left-handed coaxial transmission lines is proposed. It consists in the superposition of Split Ring Resonators with an array of concentric thin wires, emulating a thin wire media, which is in principle decoupled to the array of SRR elements. Due to decoupling of the electric response of the SRR elements and the thin wire media, it is possible to obtain an initial rejected frequency band that becomes a passband when both structures are combined. The proposed structure is schematically depicted in Figure 1. Initially, in Section 2, the frequency response of the individual structures (Split Ring Resonators and thin wire media) will be described.  Section 3 describes the combination of both in order to achieve an equivalent left-handed medium, with a characteristic pass-band response, leading to the conclusions of the paper.

## 2. Coaxial Transmission Line with Periodic Inclusions

**Figure 2:**
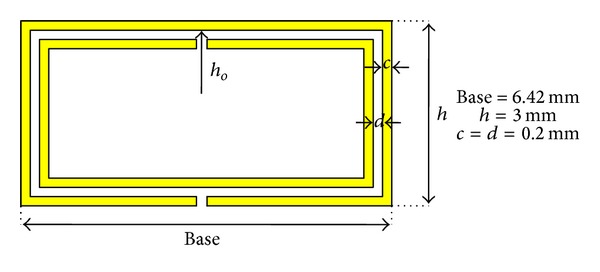
Dimensions of the SRR elements which load the host Coaxial Transmission Line. The conductor strips have a thickness of 0.1 mm.

The initial step in order to obtain frequency selective behavior is to introduce periodic inclusions in a conventional coaxial transmission line. The first step is to introduce split ring resonators embedded within the dielectric volume of the coaxial cable. The location of the SRR elements will be given by physical restrictions to fit the inclusions as well as by the configuration of the electromagnetic fields. It is a well-known fact that the coaxial transmission line supports a transverse electromagnetic mode, with E-field lines perpendicular to the central conductor spanning to the surrounding ground plane, whereas the H-field lines are solenoidal, closing around the central conductor strip. The SRR element exhibits bi-anisotropic behaviour, being the main excitation component of H-field lines which are perpendicular to the plane that contains the metallic slotted rings. Taking this fact into account, initially the most adequate position to obtain adequate excitation of the SRR elements is to place them in an axial disposition around the central conductor line, as depicted in Figure 1. The quasistatic resonance frequency can be determined by an equivalent circuit, in which the central frequency is given by an LC tank, as described in [8, 9], which is coupled to the equivalent circuit model of the transmission line. In the design proposed in this work, the dimensions of the SRR elements are given in Figure 2, which following the equivalent circuit model of the SRR exhibit a quasistatic resonance frequency in the vicinity of 2.9 GHz [8, 9]. The Coaxial Transmission Line has an inner conductor of diameter 1 mm, an outer diameter of 8.9 mm, and a dielectric of *ɛ*_*r*_ = 2.43.

**Figure 3:**
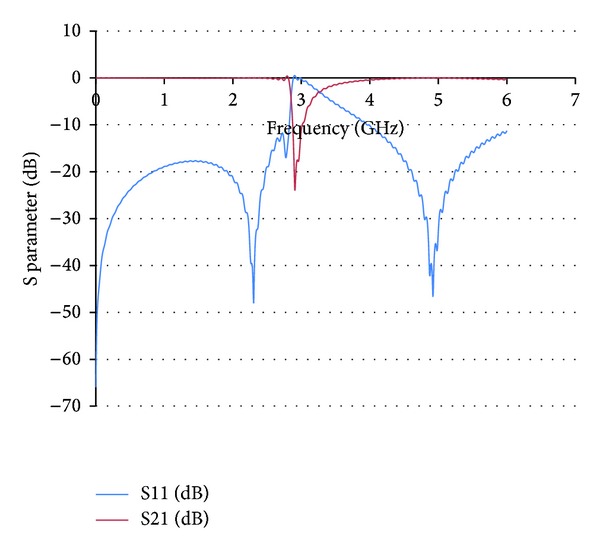
Full wave simulation results for S21 and S11 parameters in a Coaxial Transmission Line loaded with Split Ring Resonators. The quasistatic resonance frequency is approximately at 2.9 GHz, determined by the dimensions of the SRR elements embedded in the dielectric core of the Coaxial Transmission Line.

**Figure 4:**
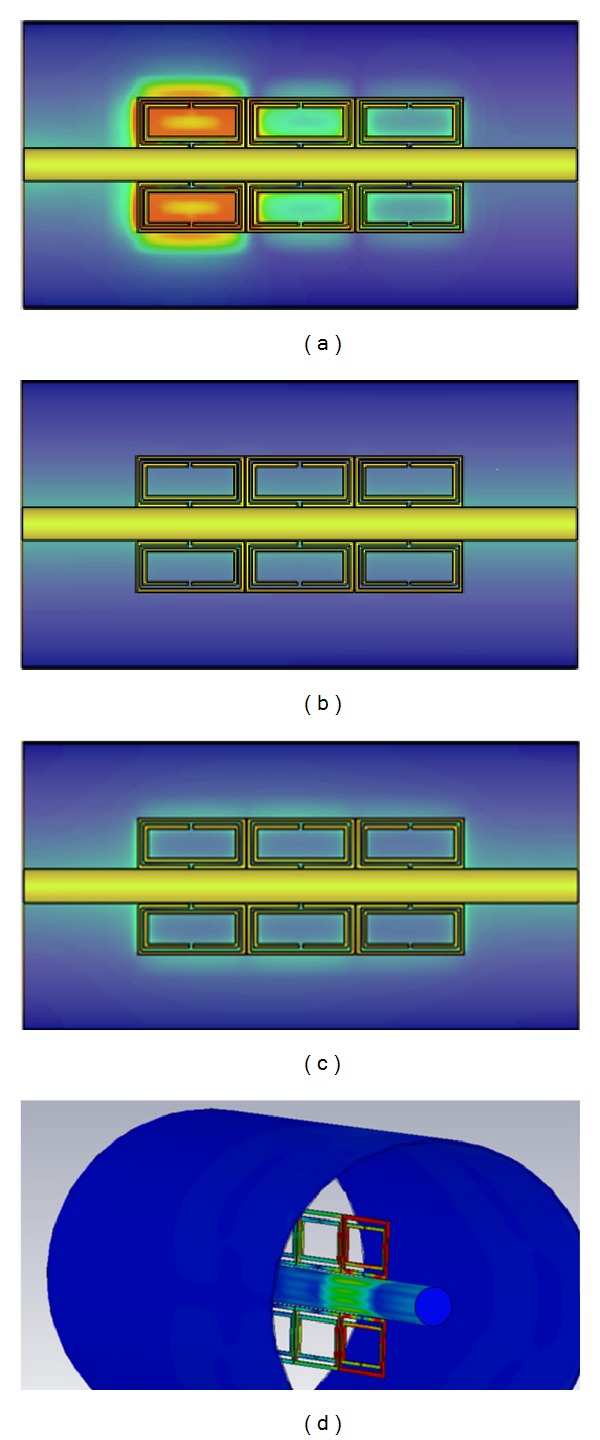
Surface current plots for the SRR loaded Coaxial Transmission Line. (a) Observation frequency of 2.9 GHz, in which quasistatic resonances can be observed within the SRR elements, (b) observation frequency of 1 GHz, (c) observation frequency of 4 GHz, and (d) volumetric representation of SRR excitation at the quasistatic resonance frequency of 2.9 GHz.

Full wave simulation results have been obtained with the aid of CST Microwave Studio for the Coaxial Transmission Line loaded with SRR elements. The host transmission line is loaded with 6 SRR elements of the dimensions previously stated in Figure 2. The overall length of the proposed prototype is 33 mm. The simulation results are shown in Figure 3, in which a rejection band in the vicinity of 2.9 GHz can be observed, clearly given by the quasistatic resonance frequency of the SRR elements. Resonances at higher frequencies (beyond the obtained simulation result) are given either by dynamic resonances of the SRR elements or by Bragg resonances due to periodic loading. This can also be observed in the surface current density plots which are depicted in Figure 4. Excitation can be seen in the SRR elements in the quasistatic resonance frequency at 2.9 GHz, whereas little or no response from the SRR elements is observed out of the resonance band. At this band, the structure can be viewed as equivalent media with a negative value of magnetic permeability *μ*.

In order to achieve a Left-Handed structure it is necessary to combine an equivalent negative *ɛ* with the equivalent negative value of *μ* which can be observed at the plasma frequency. A simple way to achieve this condition is by exciting an array of thin wires with a parallel electric field. This approach has been successfully applied in volumetric experiments, hollow metallic waveguides as well as in planar transmission lines. Following a transmission line analogy, these radial stubs behave in a similar fashion as shunt inductors, which are connected from the central conductor to the ground plane. The number of stubs as well as their separation distance determines the overall frequency response of the device, which intrinsically high pass. Full wave simulation results have been obtained for a Coaxial Transmission Line loaded with 10 radial stubs, each one of them with a diameter of 0.1 mm (Figure 6). Each set of radial stubs is separated 10 mm, in order to allocate additional SRR elements in the composite left-handed configuration that will be described in the following section. The results depicted in Figure 4 show strong rejection values at the operating frequency of the SRR elements, in the 2.9 GHz frequency region.

**Figure 5:**
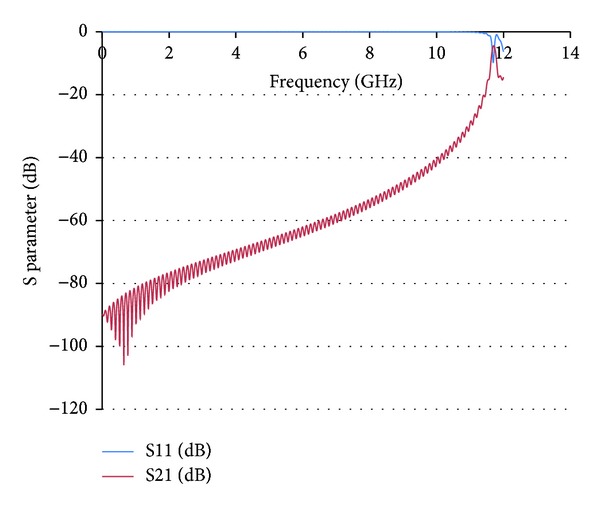
Full wave simulation results for S21 and S11 parameters in a Coaxial Transmission Line loaded with shunt radial stubs, connecting the central conductor strip with the ground plane. Due to the highly inductive nature of the radial stubs, this composite structure exhibits high rejection at the previous quasistatic resonance frequency of 2.9 GHz given by the SRR elements.

**Figure 6:**
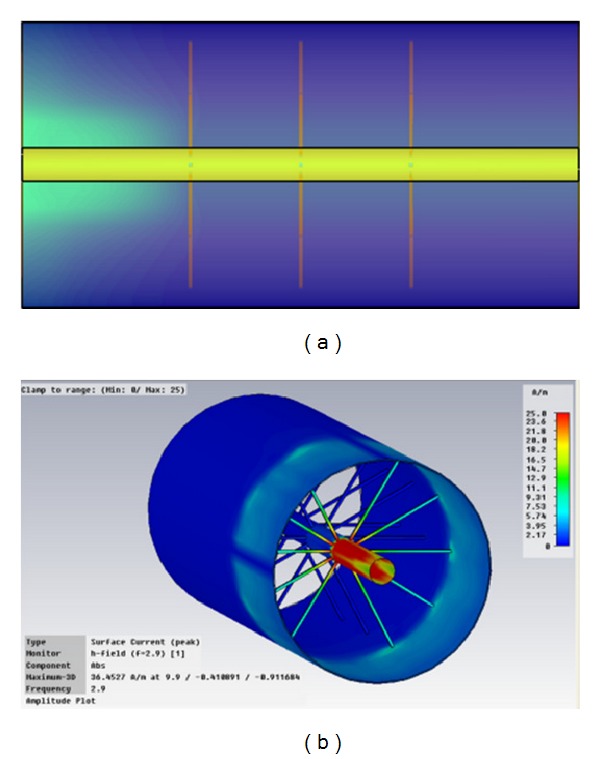
Surface current density plots for the Coaxial Transmission Line loaded with radial stubs connecting from the central conductor to the ground plane. (a) Top view of the surface current plot, (b) volumetric view of the surface current, exhibiting strong reflection at the frequency of 2.9 GHz.

The rejection given in the 2.9 GHz frequency region can be observed in the surface current plots presented in Figure 5. As it can be seen, there is very low surface current content in the output port, due to high values in the reflection coefficient determined by the S11 parameter, in line with the inductive nature of the radial stubs.

## 3. Combined Loading Elements

**Figure 7:**
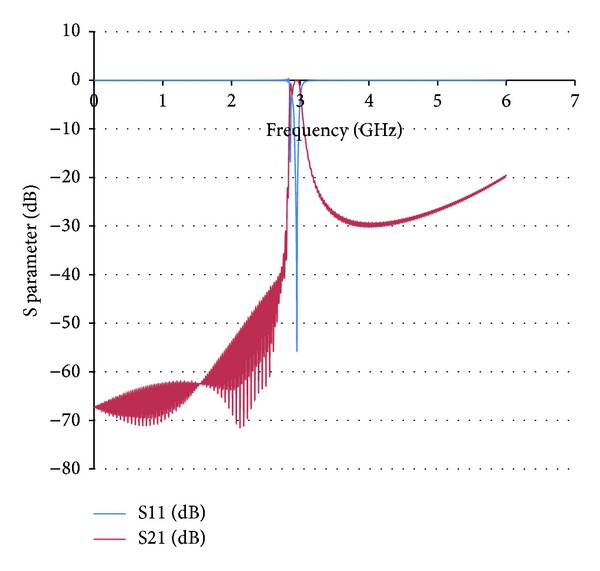
Full wave simulation results for the combined SRR + radial stub loaded Coaxial Transmission Line. In this case a passband can be observed where initially a stopband was given by each individual loading structure.

Once the equivalent value of negative *ε* can be derived from the radial stubs loading the Coaxial Transmission Line and the equivalent value of negative *μ* is achieved by introducing SRR elements, both can be now combined in a single structure in order to obtain Left-Handed behaviour. In order to enhance the frequency response of the resulting device, both the electric response (given by the radial stubs) and the magnetic response (given by the set of SRR elements) should be decoupled [8, 9]. Due to the fact that the fundamental mode in the Coaxial Transmission Line is a TEM mode, E-field components extend from the central conductor to the external ground plane conductor, perpendicular to the conductor surfaces, whereas the H-field lines close around the central conductor strip in a concentric manner. Therefore, the optimal configuration to achieve such decoupling is the arrangement depicted in Figure 1, in which the arrays of radial stubs are located within the normal plane in which the slits of the metallic rings of the SRR are located. The result of such a combined structure is a passband where previously a stopband was present, as can be seen from the full wave simulation results depicted in Figure 7.

**Figure 8:**
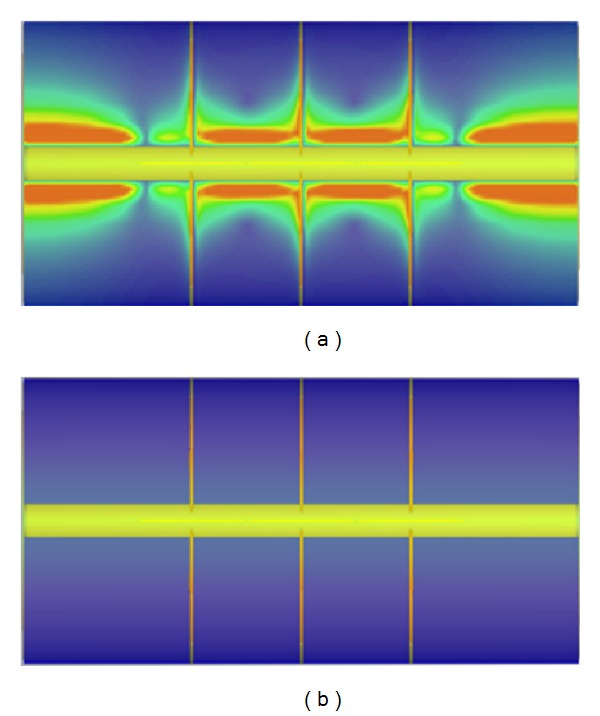
Power flow plots for the composite loaded Coaxial Transmission Line. The left image corresponds to a frequency of 2.9 GHz, whereas the right image is for a frequency of 1 GHz. As it can be seen there is high power transmission in the first case, whereas strong rejection can be observed in the second case.

**Figure 9:**
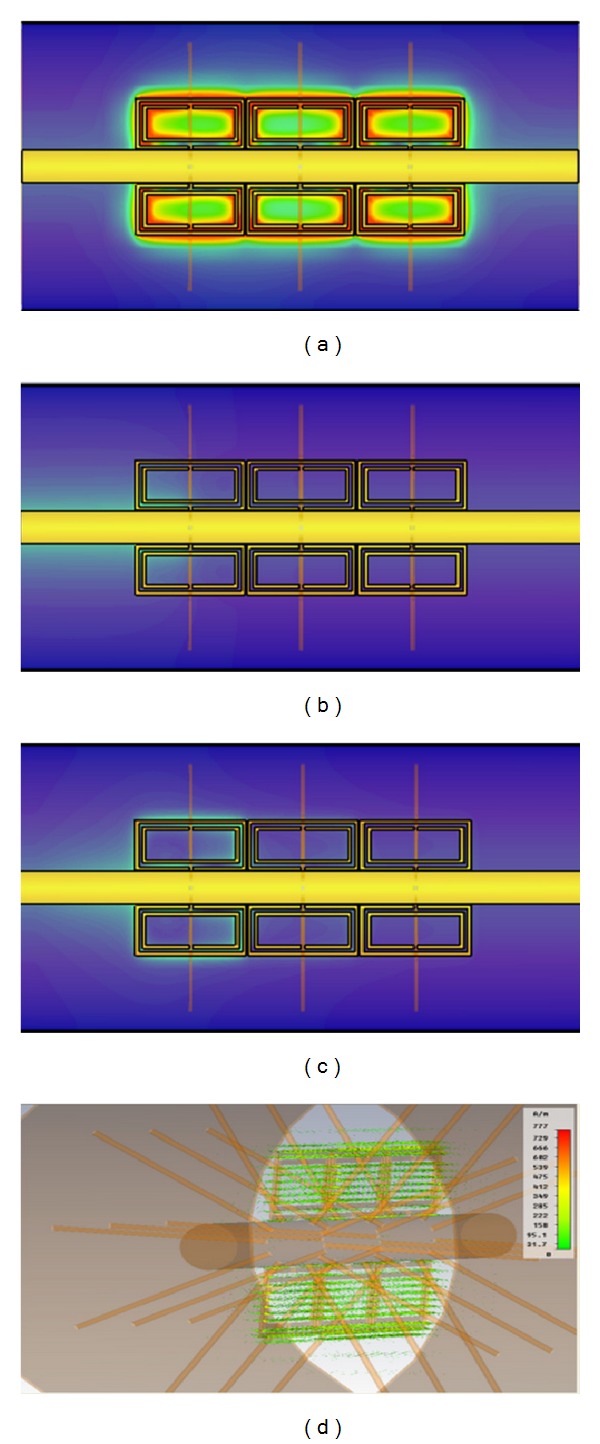
Surface current plots for the composite SRR + radial stub loaded Coaxial Transmission Line. (a) Observation frequency of 2.9 GHz, in which quasistatic resonances can be observed within the composite structure, (b) strong rejection present in the observation frequency of 1 GHz, (c) observation frequency of 4 GHz, and (d) volumetric representation of SRR excitation at the quasistatic resonance frequency of 2.9 GHz.

This passband exists due to the combined effect of the quasistatic resonance frequency of the SRR elements and the strong inductive response of the radial stubs at this frequency. Once out of the bandwidth of operation of the quasistatic resonance frequency of the SRR element, rejection is once again observed, since only reactive power can now be propagated. This can be clearly seen in the power flow plots depicted in Figure 8, as well as in the surface current density plots shown in Figure 9.

## 4. Conclusions

In this work, a novel configuration in order to achieve Left-Handed behavior in a Coaxial Transmission Line is presented. By combining a set of Split Ring Resonators and an array of radial conductor stubs, a passband where in principle a stopband should appear can be obtained. This is given by the adequate excitation of the combination of SRR elements with the radial stubs, avoiding magnetoelectric coupling. The proposed approach can be employed in order to implement frequency selective devices in coaxial technology, which can be in principle scaled in frequency in order to cover a broad range of potential applications.
